# Even High-Quality CPGs Seldom Include Implementation Strategies

**DOI:** 10.3389/fphar.2020.593894

**Published:** 2021-01-12

**Authors:** Luciana Pereira de Vasconcelos, Daniela Oliveira De Melo, Airton Tetelbom Stein, Heráclito Barbosa de Carvalho

**Affiliations:** ^1^ Department of Preventive, Medicine Medical School, São Paulo University, São Paulo, Brazil; ^2^ Department of Pharmaceutical Science, Institute of Environmental, Chemical and Pharmaceutical Sciences, Federal University of São Paulo, São Paulo, Brazil; ^3^ Department of Collective Health, Federal University of Health Sciences of Porto Alegre, São Paulo, Brazil

**Keywords:** clinical practice guideline, agree Ⅱ, appraisal (evaluation), applicability, non communicable chronic diseases, implementation tools, implementation strategies

## Abstract

**Background:** Implementation is a key step in ensuring that high-quality clinical practice guideline (CPG) recommendations are followed and have a positive impact. This step must be planned during CPG development. This study aims to inform professionals tasked with developing and implementing CPGs regarding implementation strategies and tools reported in high-quality CPGs for chronic non-communicable diseases (NCDs).

**Methods:** NCD guidelines were selected based on Appraisal of Guideline Research and Evaluation (AGREE) II assessment. CPGs with a score of ≥60% in AGREE II domains 3 (rigor of development), 5 (applicability), and 6 (editorial independence), were considered high quality. The content related to implementation was extracted from CPG full texts and complementary materials. Implementation strategies and tools were assessed and classified using Mazza taxonomy.

**Results:** Twenty high-quality CPGs were selected, most of which were developed by government institutions (16; 80%) with public funding (16; 80%); almost half (9; 45%) addressed the treatment of cardiovascular diseases. The countries with the most high-quality CPGs were the UK (6; 30%) and Colombia (5; 25%). These countries also had the highest average number of strategies, Colombia with 28 (SD = 1) distributed in all levels, and the UK with 15 (SD = 7), concentrating on professional and organizational levels. Although the content of the Colombian CPGs was similar regardless the disease, the CPGs from the UK were specific and contained data-based feedback reports and information on CPG compliance. Implementation strategies most frequently identified were at the professional level, such as distributing reference material (18; 80%) and educating groups of healthcare professionals (18; 80%). At the organizational level, the most frequent strategies involve changes in structure (15; 75%) and service delivery method (13; 65%).

**Conclusion:** Countries with established CPG programs, such as the UK and Colombia, where identified as having the highest number of high-quality CPGs, although CPG implementation content had significant differences. Among high-quality CPGs, the most common implementation strategies were at the professional and organizational levels. There is still room for improvement regarding the implementation strategies report, even among high-quality CPGs, especially concerning monitoring of implementation outcomes and selection of strategies based on relevant implementation barriers.

## Introduction

Clinical practice guidelines (CPGs) have been developed by several institutions aiming at reducing the variability in the health care procedures, as well as producing trustworthiness guidelines (Greenfield et al., 2011). Developing a high-quality CPG requires a great deal of financial and human resources and time. There is a need to involve a multidisciplinary group, including patient and methodologists, as stakeholders (Fervers et al., 2011; Burgers et al., 2012; Qaseem et al., 2012; Kristiansen et al., 2014). Implementation is a key step and must be planned during the CPG development, as suggested in the checklist proposed by Schünemann et al. (Schünemann et al., 2014). The effort and resources involved in developing a CPG of high methodological quality can be wasted if not properly implemented (Greenfield et al., 2011).

CPG quality has progressed, as shown by at least two meta-reviews (Alonso-Coello et al., 2010; Armstrong et al., 2017). Both included primary studies where CPG quality was assessed using the Appraisal of Guideline Research and Evaluation (AGREE) instrument in its first and/or second version (Alonso-Coello et al., 2010; Armstrong et al., 2017). The AGREE instrument is considered the best validated instrument for CPG quality assessment (Siering et al., 2013) and comprises the following domains: 1) scope and purpose; 2) stakeholder’s involvement; 3) rigor of development; 4) clarity of presentation; 5) applicability, and 6) editorial independence. Although CPG quality has improved in many domains, such as rigor of development, clarity, scope, and even stakeholder involvement, applicability scores remain the lowest. Another systematic review of this domain showed that scores did not improve between 2008 and 2013, remaining below other AGREE domains and only reaching a mean of 43.6%, at a scale of 0–100%, with 100% being the best (Brouwers et al., 2010; Brouwers et al., 2013; Gagliardi and Brouwers, 2015).

Although assessment of the AGREE II applicability domain helps identify implementation gaps, other aspects must be considered. In 2002, the Cochrane Effective Practice and Organization of Care (EPOC) group published a checklist to guide systematic reviews on implementation, as well as a taxonomy to guide the extraction of relevant information from implementation studies (Cochrane Effective Practice and Organisation of Care (EPOC), 2011). Based on the EPOC checklist, Mazza et al. developed a revised taxonomy with four levels (professional, financial, organizational, and regulatory) and 49 implementation strategies (Mazza et al., 2013).

Gagliard et al. (Gagliardi et al., 2016), has previously used Mazza taxonomy on randomized and non-randomized studies to assess implementation strategies that described methods used to implement new guidelines or promote compliance with guidelines on specific conditions (arthritis, colorectal cancer, diabetes, and heart failure). This study aimed to describe the strategies used and identify trends in overtime and clinical topic use, which may suggest implementation strategies that suit different barriers and circumstances. The study has shown that the most common strategies are at the professional level as education on guideline intent and benefits, reminders to professional groups about guideline intent, and provision of print material, such as summaries, algorithms, or referral forms.

Studies on the management of individual conditions evaluated the impact of specific implementation strategies (Forsetlund et al., 2009; Arditi et al., 2012; Giguère et al., 2012), but to the best of our knowledge, no study has evaluated the report of implementation strategies and tools in high-quality CPGs. Assessing implementation content in high-quality CPGs can contribute to disseminate good practices and opportunities for improvement in this area. Thus, the primary objective of this study was to inform professionals tasked with developing and implementing CPGs regarding the most frequent implementation strategies and tools reported in high-quality CPGs in chronic non-communicable diseases (NCDs) by using Mazza taxonomy (Mazza et al., 2013).

## Methods

### Selection and Description of CPGs

The CPGs were selected from a previous study from our research group, hereinafter referred to as the CHRONIDE study, where 421 CPGs on the pharmacological treatment of NCDs (cardiovascular disease, lung disease, diabetes, osteoporosis, depression, osteoarthritis dementia, gastroesophageal reflux disease, and benign prostatic hyperplasia), published in English, Spanish, or Portuguese, were assessed using the AGREE II instrument (Molino et al., 2019). The focus on NCD with pharmacological treatment was decided because of the burden of these conditions in the healthcare system and the variety of treatment options. The implementation of evidence-based CPG in NCD can improve health outcomes.

To guarantee consistency on the use of AGREE II, all documents were reviewed by three independent appraisers. They were trained following the AGREE II online training tool and pilot appraisal of two international guidelines to confirm reviewers’ understanding. The final rate for each item in the AGREE II domains (total of 23 items) was decided by consensus. Differences of ≥2, in the 7-point scale, where 1 and 7 indicate “strongly disagree” and “strongly agree,” respectively, were considered discrepant. The final score for each domain was calculated according to the instrument manual.

The AGREE II manual does not define a specific cut off or the domains considered in classifying CPGs as high quality. Thus, in this study, CPGs with scores of ≥60% in domains 3 (rigor of development), 5 (applicability), and 6 (editorial independence), were considered high quality. These domains were chosen based on other studies that considered these as the most relevant domains for assessing CPG quality (Hoffmann-Esser et al., 2017; Hoffmann-Eßer et al., 2018). By choosing the cut off of 60% in the selected three domains, we believe that the sample is composed of CPGs with more comprehensive report of implementation strategies and, at the same time, with an adequate description of development methods and disclaimer.

Two researchers extracted the following CPG data: year of publication, country, disease, and type of institution that developed the guideline (government, professional society, or university). The institution is classified as government if the CPG was developed or implemented by a government agency.

### Assessment of Implementation Strategies

To assess implementation strategies, all contents on the implementation session and tools were extracted from complete CPGs and supplementary documents and stored in an Excel® spreadsheet. Only one researcher extracted these data (LVP). This was applied to identify and categorize strategies and tools based on Mazza taxonomy (Mazza et al., 2013). The taxonomy is composed of 49 strategies divided in four levels: professional, finance, organizational, and regulatory levels with 15, 12, 18, and four strategies, respectively (Table 1).

**TABLE 1:** Representation of Mazza’s taxonomy.

Level	Strategies	
Professional	Distribute guideline materials (via hard-copy, audio-visual and/or electronic means)	
	Educate groups of health care professionals about the intent and benefit of complying with a guideline	
	Present guideline materials at meetings (including conferences, lectures, workshops or traineeships)	
	Educate individual health care professionals about the intent and benefit of complying with a guideline	
	Identify barriers to guideline implementation (including any activity aimed at identifying reasons why compliance with a guideline might not be achieved, to assist in planning strategies)	
	Feedback data and information about patients to individual health care professionals or groups to improve compliance (including clinical outcome data and information, and patient self-assessments)	
	Advertise guideline materials (including advertising via any medium, targeted advertising, personal interviews, group discussions aimed at raising awareness)	
	Provide feedback based on data and information on CPG compliance to health professionals or groups of health professionals	
	Provide feedback with patient information to healthcare professionals or groups of healthcare professionals to improve compliance	
	Provide reminders to individual health care professionals or groups about the intent and benefit of complying with a guideline (via any means)	
	Provide alerts to individual health care professionals or groups when clinical practice deviates from a guideline (via any means)	
	Recruit an opinion leader who recommends the implementation of a guideline (health care professionals must recognize the authority of the opinion leader in regard to the guideline)	
	Achieve consensus among health care professionals that the guideline is appropriate for implementation (there must be data that measures consensus)	
	Feedback information from health care professionals to individuals or groups to improve compliance (including personal testimony about the experience of implementing a guideline)	
	Other	
**Level**		**Strategies**
Financial	Healthcare professionals	Incentive applicable to a health care professional (a health care professional may receive a direct or indirect financial reward or benefit for complying with a guideline)
		Incentive applicable available to the institution (the institution or a group of health care professionals may receive a direct or indirect financial reward or benefit for complying with a guideline)
		Change in reimbursement (including any addition, subtraction or substitution of a reimbursable product or service that increases the likelihood of improved implementation)
	Patients	Incentive applicable to a patient (a patient may receive a direct or indirect financial reward or benefit if provided with care that complies with a guideline)
		Other
**Level**		**Strategies**
Organizational	Healthcare professionals	Creation of an implementation team (including creation of a multidisciplinary team of health professionals who work together on implementation)
		Reallocated roles to assist implementation (including any redistribution of roles among health professionals to facilitate implementation)
		Additional human resources provided for implementation (including increase in the number of staff and change in the type and qualifications of staff to facilitate implementation)
		Communication between distant health professionals (including establishment of any type of telecommunication link for implementation)
	Patients	Consumer feedback, suggestions and complaints (including any new process that uses information from patients to improve implementation)
		Consumer participation in governance (including any change in governance that enables patients to recommend the implementation of a guideline)
		Other
	Structural	Change in organizational structure (including any service reorganization designed to improve implementation)
		Change to the setting or site of service delivery (including any translocation of a service designed to improve implementation)
		Change in the integration of services (including change in how services are linked and integrated to improve implementation)
		Change in the method of service delivery (including any change to how a service is delivered; e.g. replacement of a traditional pharmacy with a mail order pharmacy)
		Change in the physical structure, facilities or equipment of a service (including any change to the infrastructure of a service designed to improve implementation)
		Change in quality assurance, quality improvement and/or performance measurement systems (including any quality system designed to improve implementation)
		Change in information and communication technology supporting a service (including any IT application designed and commissioned to improve implementation; e.g., computerized records, patient tracking systems, electronic referral systems, picture archiving and communication system, telehealth system, on-line text messaging)
		Change in risk management provisions (including any change in insurance cover for loss or damage to facilities, injury to staff, adverse patient outcomes and malpractice that encourages implementation)
		Other
Regulatory	Change in licensing, credentialing or accreditation of the health service and its elements (including any change that is relevant to the status, legality or reputation of the health service and its employees that increases the likelihood of implementation success)	
	Change in the ownership or affiliation (include any change which increases the likelihood of implementation success)	
	Change in legislation or regulation (include any change which enforces or mandates implementation)	
	Other	

Similarities were identified in high-quality CPGs developed in the same country; therefore, these were presented in clusters by country to summarize the findings. Codes were developed to identify CPGs based on the country of development and health condition addressed.

## Results

### Selection and Description of CPGs

Of 421 CPGs, 20 had scores of ≥60% in domains 3, 5, and 6 (Agencia de Evaluación de Tecnologías Sanitarias de Andalucía (AETSA), 2012; National Vascular Disease Prevention Alliance, 2012; Goblirsch et al., 2013; Ministerio de Salud, 2013; Ministerio de Salud y Protección Social-Colciencias, 2013a; Ministerio de Salud y Protección Social-Colciencias, 2013b; National Institute for Health and Care Excellence, 2014a; National Institute for Health and Care Excellence, 2014b; Ministerio de Salud y Protección Social-Colciencias, 2014a; Ministerio de Salud y Protección Social-Colciencias, 2014b; Ministerio de Salud y Protección Social-Colciencias, 2014a; Ministerio de Salud y Protección Social-Colciencias, 2014b; Guideline Adaptation Committee, 2016; Ministerio de Salud y Protección Social, Departamento Administrativo de Ciencia, Tecnología e Innovación - Colciencias, 2016; National Institute for Health and Care Excellence, 2016; Scottish Intercollegiate Guidelines Network (SIGN), 2016a; Scottish Intercollegiate Guidelines Network (SIGN), 2016b; Malaysian Ministry of Health, 2017a; Malaysian Ministry of Health, 2017b; National Institute for Health and Care Excellence, 2019a; National Institute for Health and Care Excellence, 2019b; National Institute for Health and Care Excellence, 2019c). These CPGs were developed in the UK (n = 6), Colombia (n = 5), Australia (n = 2), Scotland (n = 2), Malaysia (n = 2), Chile (n = 1), Spain (n = 1), and USA (n = 1). They were mostly developed by governmental institutions (15; 75%) and predominantly (11; 55%) addressed the treatment of cardiovascular diseases, as described in Table 2.

**TABLE 2:** Description of 20 high-quality clinical practice guidelines (CPGs).

CPG Code	Country	Disease	Year	Institution	Type of institution
AUScvd (National Vascular Disease Prevention Alliance, 2012)	Australia	Cardiovascular	2012	NVDPA	Government
AUSdementia (Guideline Adaptation Committee, 2016)	Australia	Dementia	2016	NHMRC	Government
CHLasthma (Ministerio de Salud, 2013)	Chile	Asthma	2013	SSP	Government
COLdepr (Ministerio de Salud y Protección Social-Colciencias, 2013a)	Colombia	Depression	2013	IETS	Government
COLcopd (Ministerio de Salud y Protección Social-Colciencias, 2014a)	Colombia	Chronic obstructive pulmonary disease	2014	PUJ	University
COLdld (Ministerio de Salud y Protección Social-Colciencias, 2014b)	Colombia	Dyslipidemia	2014	IETS	Government
COLdm (Ministerio de Salud y Protección Social, Departamento Administrativo de Ciencia, Tecnología e Innovación-Colciencias, 2016)	Colombia	Diabetes mellitus	2016	PUJ	University
COLhbp (Ministerio de Salud y Protección Social-Colciencias, 2013b)	Colombia	High blood pressure	2013	IETS	Government
SP0 (Agencia de Evaluación de Tecnologías Sanitarias de Andalucía (AETSA), 2012)	Spain	Atrial fibrillation	2012	AETSA	Government
SCOasthma (Scottish Intercollegiate Guidelines Network (SIGN), 2016a)	Scotland	Asthma	2016	SIGN	Government
SCOchf (Scottish Intercollegiate Guidelines Network (SIGN), 2016b)	Scotland	Chronic heart failure	2016	SIGN	Government
USAcvd (Goblirsch et al., 2013)	United States	Stable coronary artery disease	2013	ICSI	Professional society
MYSdld (Malaysian Ministry of Health, 2017b)	Malaysia	Dyslipidemia	2017	NHAM	Professional society
MYScvd (Malaysian Ministry of Health, 2017a)	Malaysia	Cardiovascular	2017	NHAM	Professional society
UKaf (National Institute for Health and Care Excellence, 2014a)	United Kingdom	Atrial fibrillation	2013	NICE	Government
UKgord (National Institute for Health and Care Excellence, 2014b)	United Kingdom	Gastroesophageal reflux	2014	NICE	Government
UKhbp (National Institute for Health and Care Excellence, 2019a)	United Kingdom	High blood pressure	2016	NICE	Government
UKcvd (National Institute for Health and Care Excellence, 2016)	United Kingdom	Stable angina: Management	2016	NICE	Government
UKcopd (National Institute for Health and Care Excellence, 2019b)	United Kingdom	Chronic obstructive pulmonary disease	2016	NICE	Government
UKdm (National Institute for Health and Care Excellence, 2019c)	United Kingdom	Diabetes mellitus	2017	NICE	Government

NVDPA, National Vascular disease Prevention Alliance; NHMRC, National Health and Medical Research Council; SSP, Subsecretaria de Salud Pública; IETS, Instituto de Evaluación Tecnológica en Salud; PUJ, Pontificia Universidad Javeriana; SIGN, Scottish Intercollegiate Guidelines Network; AETSA, Agencia de Evaluación de Tecnologías Sanitarias de Andalucía; ICSI, Institute for Clinical Systems Improvement; NHAM, National Heart Association of Malaysia; NICE, National Institute for Health and Care Excellence.

### Implementation Strategies

**FIGURE 1:**
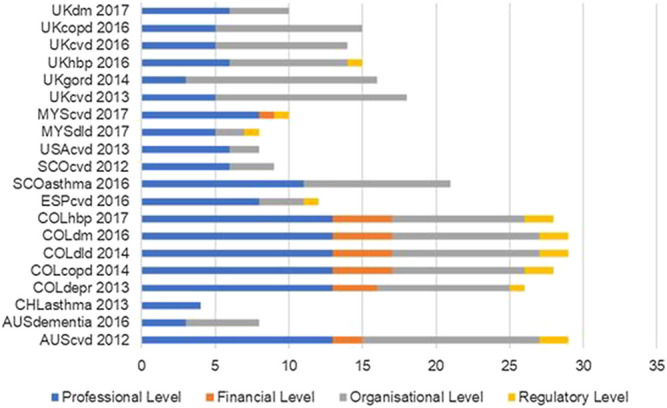
Number of implementation strategies per high-quality CPG according to Mazza taxonomy.

The largest number of strategies in a single CPG was 29 from 49 strategies described in the Mazza taxonomy (Mazza et al., 2013), as shown in Figure 1. The mean of strategies per CPG was 16.8 ± 8.5, with more strategies at the professional level. Only CPGs from Colombia and one from Australia (AUScvd) mentioned strategies at all four levels of the Mazza taxonomy.

The vast majority of implementation strategies in the CPGs from Colombia were identical, indicating that the same outline had been applied to describe the implementation strategies independent of the specific health condition addressed in each CPG.

The implementation strategies and tools in six CPGs from the UK were specific to each disease. All UK CPGs contain implementation priorities and tools to measure implementation results. The National Institute of Clinical Excellence (NICE) impact reports are comprehensive tools wherein indicators mentioned in the CPG are measured, and improvement opportunities are discussed.

The content and tools related to professional-level strategies were standard, for example health professional education strategies and short guideline versions aimed at physicians. Even in CPGs that include implementation barriers identification as strategy, the relationship between strategies and such barriers was not clearly stated. Although 10 of these 20 CPGs mention the establishment of implementation teams, it was unclear which professionals should take part in these teams or what training is needed.

### Implementation Strategies and Tools Highlights

All CPGs from Colombia (Qaseem et al., 2012), one from Scotland (SCOasthma), and one from Australia (AUScvd) included most of the 15 possible professional-level strategies. The most frequent strategies related to the distribution of guideline materials and healthcare professional education is shown in Table 3.

**TABLE 3:** Strategies highlights per level.

Level	Professional	Financial	Organizacional	Regulatory
Strategies	Distribute guideline materials (18, 90%) - educate groups of health care professionals about the intent and benefit of complying with a guideline (18, 90%)	The CPGs from Colombia are the ones with more strategies related: 4 CPGs (COLcopd, COLdld, COLdm, COLhbp) contain 4 strategies and 1 (COLdepr) contain 3 strategies - the following strategies were not identified in any of the 20 CPGs: Grant or allowance provided to a health care professional, grant or allowance provided to the institution, penalty applicable to a health care professional, penalty applicable to the institution	Only one CPG, the SCOaf2012, mentions distance communication between health professionals and patients aiming at enhancing implementation-none of the 20 CPGs provides any guidance on health worker satisfaction for increasing commitment and satisfaction with the implementation of the CPG	4 of the 5 CPG from Colombia (COLcopd, COLdld, COLdm, COLhbp) contain 2 strategies at this level and 1 (COLdepr) contain 1 strategy - change in licensing, credentialing or accreditation of the health service and its elements, was a strategy more frequent, identified in 8 (40%) CPGs, including the 5 CPG from Colombia
			*Structural Category*-change in organizational structure (15; 75%),-change in the method of service delivery (13; 65%)-change in service integration (13; 65%). Health professional category	
			*Health professional category*-creation of an implementation team (10; 50%)	
			*Patient category*-consumer feedback (11; 55%)	

In general, strategies that covered financial aspects were minimally explored even in high-quality CPGs, and in 13 (65%) CPGs no financial-level strategy had been described.

At the organizational level, CPGs from Colombia, UK, and Australia had the most strategies identified, especially in the structural category. Only one CPG, the SCOaf2012, mentioned distance communication between health professionals and patients as a means of enhancing implementation. None of the CPGs analyzed provided any guidance on how to measure the satisfaction and commitment of professionals with the implementation of the document.

Four of the five CPGs from Colombia (COLcopd, COLdld, COLdm, and COLhbp) contained two strategies at the regulatory level, and one (COLdepr) contained one strategy. Changes in licensing, credentialing, or accreditation of healthcare services were the most frequent strategy, being identified in eight (40%) CPGs, including the five CPGs from Colombia. No implementation tool had been identified at the regulatory level (Table 4).

**TABLE 4:** Tools highlights per level.

Level	Professional	Financial	Organizacional	Regulatory
Tools	Clinical algorithms-summarized versions of CPGs for physicians - materials for classes and presentations of CPGs	None implementation tool was identified	The largest number of tools at organizational level was found in CPGs from United Kingdom.	None implementation tool was identified
			Patient’s category-version of the CPGs written in lay language for patients and caregivers	
			Structural category-spreadsheets with implementation tracking indicators,- implementation plans - spreadsheets for initial assessment of clinical parameters	

## Discussion

Most of the high-quality CPGs were developed in countries with existing CPG development programs, such as the UK and Colombia, consistent with previous studies (Burgers et al., 2003; Molino et al., 2019). Our results showed that CPGs from Colombia contained the most implementation strategies, including strategies at all levels. Although the Colombian CPG development program stood out in producing high-quality CPGs and implementation strategies, it should be highlighted that the content devoted to implementation was similar regardless of disease. By contrast, the strategies and tools were specific to each disease in CPGs developed in the UK, and implementation was expected to be more effective. A Cochrane systematic review concluded that tailored implementation strategies were more effective than general ones (Baker et al., 2015). Therefore, analysis of the number of implementation strategies is not sufficient to fully understand CPG implementation. The qualitative assessment of the strategies is relevant to understand how CPG developers and relevant institutions plan implementation. Although the number of strategies varies across high-quality CPGs, strategies at the professional level are predominant, whereas financial and regulatory level strategies are reported less frequently.

In the CHRONIDE study, Europe had the second highest number of CPGs (124; 30%) (Molino et al., 2019), and in the current study, Europe had contributed with half of the high-quality CPGs (10; 50%). It is worth noting that only CPGs available in English, Spanish, or Portuguese were included in our sample, which may have led to the exclusion of CPGs from other WHO countries, such as Germany and France, potentially limiting the generalization of findings (Panteli et al., 2019). There were 6 (30%) out of the 20 high-quality CPG that had been developed by NICE, which is based in the UK.

Since 2009, NICE was responsible for developing and maintaining quality indicators within the Quality Outcomes Framework. Concerning implementation tools, CPGs from the UK are highly regarded for tools, such as the NICEimpact for cardiovascular disease prevention and NICEimpact for diabetes, with data based on feedback reports and information on CPG compliance (National Institute for Health and Care Excellence, 2018). These reports aim to evaluate the acceptance of CPG recommendations developed by the NICE and measure their impact on health outcomes. Thus, NICE develops and evaluates CPG results as part of a structured program, which may have contributed to CPGs from the UK being considered high quality (National Institute For Health And Care Excellence, 2020).

Although Latin America was the fourth region with the most CPGs (54; 13%) in the CHRONIDE study (Molino et al., 2019), it had the highest proportion of high-quality CPGs (6; 11%). However, it should be noted that the results can be largely attributed to Colombia (5; 25%). Most Colombian CPGs were developed by the Institute of Health Technology Assessment and Technology (IETS), established in 2012 by the Colombian Administrative Department of Science, Technology, and Innovation (COLCIENCIAS). In addition to developing CPGs, IETS is also responsible for developing implementation strategies and tools, which may contribute to their quality (IETS, 2020). The fact that the Colombian’s CPG contained the largest number of implementation strategies does not necessarily mean that the Colombian health system has the best health outcomes. The Colombian Health-Related Sustainable Development Goals (SDGs) Index was 58.8 and 65.8 in 2011 and 2017, respectively, and despite this improvement, it is still behind countries, such as the UK, with an index of 77.0 and 80.4 in 2011 and 2017, respectively. This index evaluates how far countries are from the United Nations SDGs created to encourage improvements in health, equity, and well-being by 2030 (Institute for Health Metrics and Evaluation, 2020).

As expected, although the United States and Canada were responsible for the largest number of CPG evaluated in the CHRONIDE study (129; 31%) (Molino et al., 2019), only one of these was considered high quality in the present study. These results might be due to the structure of their healthcare systems. Despite the USA Institute of Medicine developing the Clinical Guidelines We Can Trust report in 2011 (Greenfield et al., 2011) with standards for developing trustworthy guidelines and the existence of other important CPG sources in the country, such as Emergency Care Research Institute repository of CPGs, there is no national CPG development program, such as NICE. In Canada, the Canadian Agency for Drugs and Technologies in Health is responsible for evaluating health technologies and making recommendations; however, the provinces are autonomous, and CPGs are usually developed by medical societies (Molino et al., 2019).

The implementation strategies most frequently found among the high-quality CPGs were at the professional level, consisting mainly of the distribution of reference material, individual and group education of healthcare professionals, and presentation of CPG material at meetings. This is consistent with reports in previous studies (Pantoja et al., 2017; Tomasone et al., 2020). Clinical algorithms, CPG versions directed at physicians, and material for classes, and presentations on CPGs were commonly found at the professional level. Such tools mainly aim to disseminate CPGs to healthcare professionals, and although they can be seen as a basic and essential strategy, studies show these strategies alone are insufficient (Francke et al., 2008), with the impact of educational meetings, for example, considered low (Forsetlund et al., 2009).

Financial and regulatory level strategies were not mentioned in the UK guidelines, possibly due to the healthcare system structure, with funding based on nationwide collection and allocation of resources by the Department of Health and Social Care (Nicoletti and Faria, 2017). Therefore, strategies, such as financial incentives or changes in licensing, accreditation, or credentialing of healthcare services and its elements, are not the responsibility of NICE (National Institute For Health And Care Excellence, 2020). This does not mean that financial incentive strategies do not exist in the UK, but that they were not found in CPGs and supplementary documents provided by NICE. Therefore, to fully understand CPG implementation programs in a specific country, understanding its healthcare system’s organization, funding, and social determinants is necessary. This is an important insight for future studies of CPG implementation best practices.

In the structural category of organizational level, strategies related to changes in organizational structure were the most frequent (15; 75%), followed by changes in the method of service delivery (13; 65%), and integration between health services (13; 65%), especially in CPGs from Australia, Colombia, and the UK. In the health professional’s category, strategies, such as the establishment of an implementation team (10; 50%) and relocation of roles (9; 45%) were the most frequent. Although CPGs suggested that implementation teams should be established, they did not define appropriate team composition or training. Studies show that although implementation of science training programs exists, few are intended for the practitioners responsible for planning and carrying out implementation strategies. These studies call such professional implementers, “implementation champions,” “knowledge brokers,” and “facilitators” (Proctor et al., 2019).

None of high-quality CPGs clearly addressed the relationship between implementation strategies and possible barriers to implementation. This aspect should be further explored by institutions developing CPGs as strategies aimed at overcoming such barriers can increase their effectiveness (Jäger et al., 2016). Most institutions do not routinely monitor the implementation of CPGs. Publications reporting surveillance data, such as the NICEimpact reports, could be helpful. Other types of reports, such as those using structured interviews with healthcare professionals on implementation strategies, may provide important information on preferred and most effective strategies (Adams et al., 2018).

The identification of barriers to guideline implementation is a fundamental step toward the selection of appropriate implementation strategies and is presented in 15 of the 20 CPGs. However, these CPGs did not provide any guidance on how to identify and solve the barriers. Brainstorming is a technique for identifying barriers that has no cost and can be used in chronic diseases (Krause et al., 2014). Including explanations and advice on this strategy in CPGs might help institutions identify relevant barriers and choose the most appropriate implementation strategies for their context. Therefore, the association between implementation strategies and their barriers was unclear in these high-quality CPGs. This is consistent with results found in a scoping review on trends in CPG implementation, wherein the authors show that the process of defining strategies based on relevant barriers did not change over time, despite increased awareness of its relevance to health outcomes, and the publication of several models, theories, taxonomies, and frameworks aimed at improving implementation (Gagliardi and Alhabib, 2015).

Because some studies show that the use of implementation strategies, such as alerts and reminders, can increase adherence to CPG recommendations, the use of these strategies should be encouraged. (Flodgren et al., 2016; Stein et al., 2018). However, such interventions were uncommon in the CPGs analyzed. The most common strategy (13; 65%) was the direct provision of patient data and feedback to healthcare professional. CPGs from Colombia and Australia frequently described the use of alerts and feedback regarding compliance or deviation CPGs. Organizational level tools related to patient category, such as plain-language CPG directed at patients and caregivers, were very frequent.

Decision support tools such as decision aids and option grid were identified in only three CPGs in the UK. The adoption of decision support tools based on good communication, patient autonomy, and active involvement in treatment choice has been reported in the literature as fundamental for the progression of the healthcare systems toward a value-based outlook (Bae, 2017). Although such tools are well-known, caution should be taken in their development to ensure that they are reliable. It is important to avoid scientific, financial, and ideological interests outweighing the benefits of increased patient participation in decision making by balancing risks and benefits and considering patient values and preferences (Moore et al., 2017).

The CPGs described implementation strategies at different levels of the Mazza taxonomy; however, none used the multifaceted approach, characterized by the simultaneous use of several implementation strategies (Suman et al., 2016). Based on the contents of these CPGs, it is not possible to affirm that countries are following this approach. Although publications, such as the BRIDGE study (Berwanger et al., 2012), show that the multifaceted approach increases CPG adherence, other studies report that multifaceted strategies do not change professional behavior toward CPG adherence (Suman et al., 2016).

The main limitations of this study are the language restriction and the review of published documents only, which may exclude strategies that influence implementation but are used internally by institutions as audit reports for example. Although AGREE II is a highly accepted instrument for assessing the quality of CPG, it has inherent limitations such as some degree of subjectivity, which has been reduced by the participation of three trained appraisers. Regarding the influence of the chosen 60% cut-off, although it is directly related to the number of selected CPG, this cut off has been used in most studies that use AGREE II to identify high-quality CPG. The data extraction and classification of strategies and tools were performed by only one researcher. For more complete view of all implementation strategies, future studies may include the assessment of all institutions’ policies and procedures, which would imply active participation of institutions in information collection. Although the number of high-quality CPGs was small, the institutions that developed them are incorporating implementation strategies in their documents, as recommended by some authors (Gagliardi et al., 2015). However guidelines developers and stakeholders can improve the reporting of implementation by considering the following: report tailored strategies, assess barriers, describe specific strategies to overcome those barriers, consider strategies in all four levels of Mazza taxonomy to avoid the tendency of focusing on the professional level, and establish monitoring of adherence of CPG recommendations with assessment of outcomes. The GRADE working group advises that institutions and teams responsible for CPG development must consider the aspects influencing the decision to adopt recommendations in a planned, detailed, and clear way (Alonso-Coello et al., 2016).

## Conclusion

This was the first assessment of implementation strategies in high-quality CPGs using the Mazza taxonomy. This tool was important in identifying the main strategies included in CPGs. However, the qualitative analysis of CPG and supplementary material was essential to highlight the implementation gaps where institutions should focus for improvement.

Although Colombia CPGs presented a large quantity of strategies, these were not disease specific, whereas implementation strategies and tools were specific for each disease in UK CPGs. These differences can be explained, at least partially, by the maturity of these countries’ healthcare systems and CPG development programs. Therefore, implementation studies should include a critical analysis of CPG content, as well as health policies and procedures of institutions and countries in addition to the number of strategies.

The most implementation strategies specified in these CPGs were at the professional and organizational levels, mainly consisting of strategies for disseminating CPGs among healthcare professionals and patients. There is still room for improvement regarding the implementation strategies report, even among high-quality CPGs, especially concerning monitoring of implementation outcomes and selection of strategies based on relevant implementation barriers.

## Data Availability Statement

The original contributions presented in the study are included in the article, further inquiries can be directed to the corresponding author.

## Ethics Statement

This study was assessed and approved by the Ethics Committee at São Paulo Medicine University on December 12, 2018.

## Author Contributions

LV collected, analyzed, and interpreted the data regarding implementation strategies in high-quality CPG. DM and AS participated in data interpretation and contributed in writing the manuscript, and HC substantively revised this work. All authors read and approved the final manuscript.

## Conflict of Interest

The authors declare that the research was conducted in the absence of any commercial or financial relationships that could be construed as a potential conflict of interest.

The reviewer SNS declared a past collaboration with one of the authors ATS to the handling editor.

## Glossary

AETSA: agencia de evaluación de tecnologías sanitarias de andalucía
af: atrial fibrillation
AGREE: appraisal of guideline research and evaluation
AUST: australia
chf: chronic heart failure
CHI: Chile
Col: Colombia
COLCIENCIAS: Colombian administrative department of science, technology and innovation
copd: chronic obstructive pulmonary disease
CPGs: clinical practice guidelines
cvd: cardiovascular disease
depr: depression
DH: department of health
dld: dyslipidemia
dm: diabetes mellitus
EPOC: Effective Practice and Organization of Care
ESP: Spain
gord: gastroesophageal reflux disease
hbp: high blood pressure
ICSI: institute for clinical systems improvement
IETS: institute of health technology assessment and technology
MYS: Malaysia
NCD: non-communicable diseases
NHAM: national heart association of Malaysia
NHMRC: national health and medical research council
NICE: national institute for health and care excellence
NVDPA: national vascular disease prevention alliance
PHC: primary health care
PUJ: pontificia universidad javeriana
SCO: Scotland
SIGN: scottish intercollegiate guidelines network
SSP: subsecretaria de salud pública
StaRI: standards for reporting implementation studies (stari) statement
UK: United Kingdom
USA: United States of America
